# The signal pathways and treatment of cytokine storm in COVID-19

**DOI:** 10.1038/s41392-021-00679-0

**Published:** 2021-07-07

**Authors:** Lan Yang, Xueru Xie, Zikun Tu, Jinrong Fu, Damo Xu, Yufeng Zhou

**Affiliations:** 1 Institute of Pediatrics, Children’s Hospital of Fudan University, National Children’s Medical Center, and the Shanghai Key Laboratory of Medical Epigenetics, International Co-laboratory of Medical Epigenetics and Metabolism, Ministry of Science and Technology, Institutes of Biomedical Sciences, Fudan University, Shanghai, China; 2 National Health Commission (NHC) Key Laboratory of Neonatal Diseases, Fudan University, Shanghai, China; 3 General Department, Children’s Hospital of Fudan University, Shanghai, China; 4 State Key Laboratory of Respiratory Disease for Allergy at Shenzhen University, Shenzhen Key Laboratory of Allergy and Immunology, Shenzhen University School of Medicine, Shenzhen, China; 5 Institute of Infection, Immunity and Inflammation, University of Glasgow, Glasgow, UK

**Keywords:** Infectious diseases, Infectious diseases

## Abstract

The Coronavirus Disease 2019 (COVID-19) pandemic has become a global crisis and is more devastating than any other previous infectious disease. It has affected a significant proportion of the global population both physically and mentally, and destroyed businesses and societies. Current evidence suggested that immunopathology may be responsible for COVID-19 pathogenesis, including lymphopenia, neutrophilia, dysregulation of monocytes and macrophages, reduced or delayed type I interferon (IFN-I) response, antibody-dependent enhancement, and especially, cytokine storm (CS). The CS is characterized by hyperproduction of an array of pro-inflammatory cytokines and is closely associated with poor prognosis. These excessively secreted pro-inflammatory cytokines initiate different inflammatory signaling pathways via their receptors on immune and tissue cells, resulting in complicated medical symptoms including fever, capillary leak syndrome, disseminated intravascular coagulation, acute respiratory distress syndrome, and multiorgan failure, ultimately leading to death in the most severe cases. Therefore, it is clinically important to understand the initiation and signaling pathways of CS to develop more effective treatment strategies for COVID-19. Herein, we discuss the latest developments in the immunopathological characteristics of COVID-19 and focus on CS including the current research status of the different cytokines involved. We also discuss the induction, function, downstream signaling, and existing and potential interventions for targeting these cytokines or related signal pathways. We believe that a comprehensive understanding of CS in COVID-19 will help to develop better strategies to effectively control immunopathology in this disease and other infectious and inflammatory diseases.

## Introduction

**Fig. 1:**
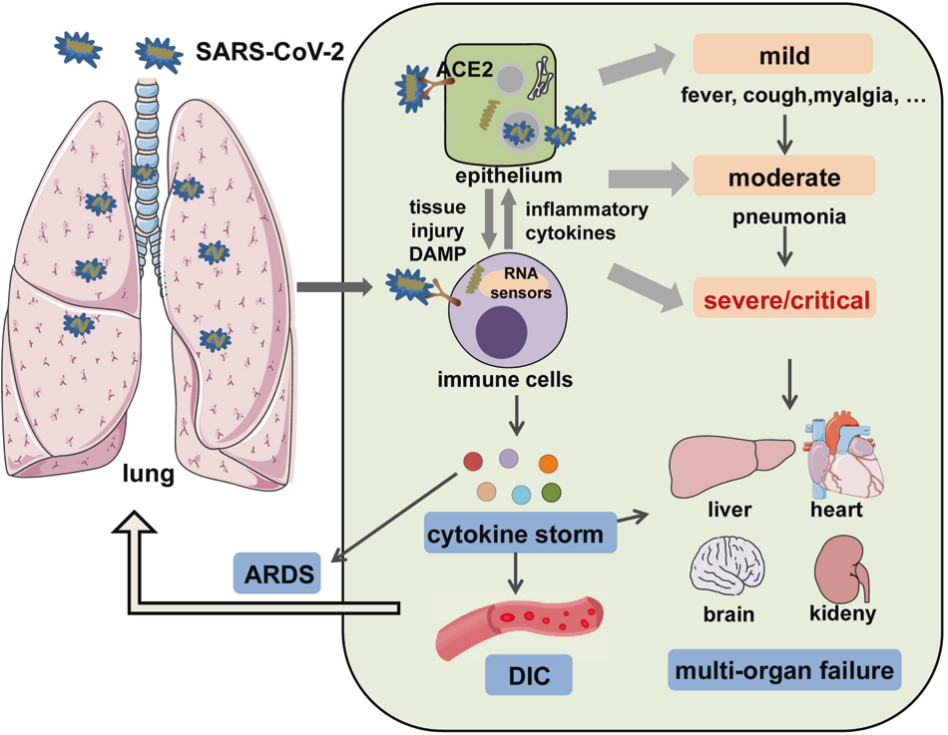
A systemic clinical manifestations of COVID-19. SARS-CoV-2 infects airway epithelial cells or immune cells via binding to ACE2 receptors, causing tissue damage and release of DAMPs, as well as production of inflammatory cytokines by epithelial cells and immune cells. Then, the crosstalk between epithelial cells and immune cells leads to a wide range of clinical manifestations, from mild forms (e.g., fever, cough, and myalgia); to moderate forms requiring hospitalization (pneumonia and localized inflammation); to severe/critical forms with a fatal outcome that are manifested as pneumonia, ARDS, DIC, CS, and multiorgan failure. DAMP danger-associated molecular pattern, ARDS acute respiratory distress syndrome, DIC disseminated intravascular coagulation

Coronavirus Disease 2019 (COVID-19) caused by severe acute respiratory syndrome coronavirus 2 (SARS-CoV-2) rapidly spread worldwide and was declared a pandemic in early 2020. COVID-19 destroyed people’s mental and physical health and staggered global economic growth. As of May 18, 2021, 163 million infections, including 3.38 million deaths, have been recorded (source: World Health Organization). SARS-CoV-2 invades the host by virtue of angiotensin-converting enzyme 2 (ACE2) receptors broadly distributed on various tissues and immune cells.^1–5^ The virus can cause a wide range of clinical manifestations from mild forms such as fever, cough, and myalgia to moderate forms requiring hospitalization (pneumonia and localized inflammation) to severe/critical forms with fatal outcomes.^6,7^ Severe or critical infection often manifests as pneumonia,^8,9^ disseminated intravascular coagulation (DIC), acute respiratory distress syndrome (ARDS), low blood pressure, and multiorgan failure (Fig. 1).^9–11^

Several lines of evidence have shown that immunopathological damage may be responsible for the deterioration of COVID-19. Particularly, multiple studies have reported that highly elevated levels of pro-inflammatory cytokines are produced during the crosstalk between epithelial cells and immune cells in COVID-19, which has linked the *cytokine storm* (CS) with the severe complications and poor outcomes in this infection.^12–14^

CS is a fast-developing, life-threatening, clinical condition in which the overproduction of inflammatory cytokines and excessive activation of immune cells lead to complicated medical syndromes from a persistent fever, nonspecific muscle pain, and hypotension, to capillary leak syndrome, DIC, ARDS, hemophagocytic lymphohistiocytosis (HLH), multiorgan failure, and death if treatment is not adequate.^15^ Therefore, the timing of diagnosis and treatment of CS could be life-saving. The term CS was first used in 1993 in graft-versus-host disease,^16^ and later, in many inflammatory diseases such as autoimmune conditions, organ transplantation, cancer chimeric antigen receptor (CAR) T cell therapy, and, most recently, in COVID-19.^17–23^ However, the profile and causative effect of CS in different conditions can greatly vary. Thus far, precise diagnosis and treatment guidelines for CS in most of the conditions are lacking. Understanding the definite alterations and pathogenic roles of individual cytokines involved in the COVID-19-related CS (COVID-CS) is hence extremely important for the development of precise diagnosis and effective treatment.

Although some aspects of this topic have been partly reviewed previously, a comprehensive view of COVID-CS to facilitate its diagnosis and treatment is still lacking with unmet clinical needs. Herein, we provide an updated and full scenario of COVID-CS from basic research to clinical diagnosis, treatment, and trials. Initially, we discuss the currently identified immunopathological features of COVID-19, especially the CS; its mechanism of action and differences with respect to CS in other disease conditions; and individual cytokines involved in the COVID-CS including their pathological role, downstream signaling, and existing interventions. In addition, the challenges and prospects in the diagnosis and treatment of COVID-CS are also discussed.

## The immunopathology of COVID-19

**Fig. 2:**
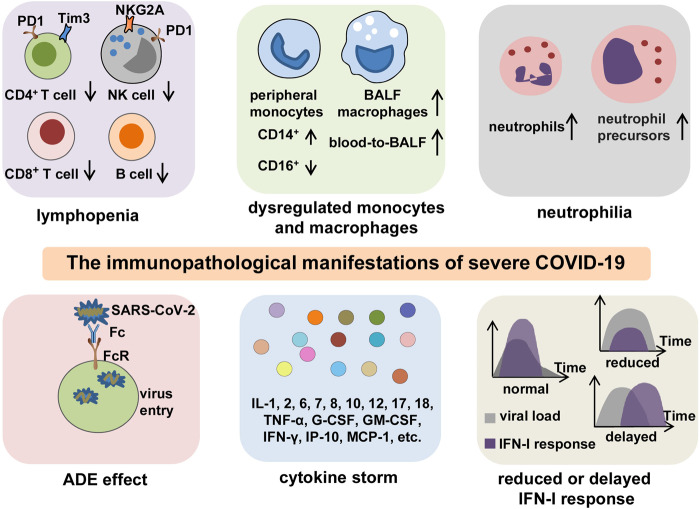
The key immunopathology of severe COVID-19. The immunopathological manifestations of COVID-19 include lymphopenia, dysregulation of monocytes and macrophages, neutrophilia, ADE, reduced or delayed IFN-I response, and CS. Lymphopenia is commonly observed in severe COVID-19. In addition to decreased counts, lymphocytes often exhibit exhaustion phenotypes with the expression of higher levels of exhaustion markers PD-1, Tim-3, or NKG2A. Peripheral monocytes present a phenotype shift from CD16^+^ to CD14^+^, and BALF macrophages are increased with a blood-to-BALF transition course. Neutrophil counts are increased with the presence of neutrophil precursors in peripheral blood, especially in patients with severe COVID-19. The possible existence of ADE enhances the entry of SARS-CoV-2 into cells through interaction between Fc regions and Fc receptors, leading to the aggravation of COVID-19. A CS is characterized by highly elevated levels of pro-inflammatory mediators and is a particularly central feature for poor outcomes in patients with severe or critical infection. Reduced or delayed IFN-I response impedes viral clearance and induces paradoxical hyperinflammation, thus leading to the deterioration of prognosis in COVID-19 patients. BALF bronchoalveolar lavage fluid, ADE antibody-dependent enhancement

In general, patients with COVID-19 present with an abnormal immune landscape, characterized by overactivated inflammatory, innate immune response, and impaired protective, adaptive immune response. This is primarily responsible for the immunopathology of severe COVID-19. Thus far, evidence from both clinical trials and basic research has revealed several key features of immunopathology in severe COVID-19, including lymphopenia, antibody-dependent enhancement (ADE), neutrophilia, dysregulation of monocytes and macrophages, reduced or delayed type I interferon (IFN-I) response, and CS (Fig. 2).

### Lymphopenia

Lymphopenia was commonly found in COVID-19 patients^10,11^ and is closely correlated with the illness severity.^24,25^ Laboratory results showed that the counts and percentages of lymphocytes including CD4^+^ T, CD8^+^ cytotoxic T,^26,27^ natural killer (NK),^24^ and B cells^25^ were all reduced in COVID-19 patients.^28–31^ Evidence from single-cell sequencing,^32^ flow cytometry,^33^ and nonhuman primate models of COVID-19^34^ confirmed the involvement of lymphopenia to the maximum extent. In addition, T cells exhibited exhaustion phenotypes with the expression of higher levels of exhaustion markers including programmed cell death protein-1 (PD-1) and T cell immunoglobulin and mucin domain-3, suggesting that these T cells may have regulatory activities.^26,27^ Other investigations showed that the PD-1- or NK group 2 member A-positive NK cells were also significantly increased in the peripheral blood of COVID-19 patients compared to healthy controls.^35,36^

Several mechanisms may contribute to SARS-CoV-2-induced depletion and exhaustion of lymphocytes: (1) SARS-CoV-2 may directly infect T cells via ACE2 receptor expressed on T cells,^37^ which results in T cell death;^38,39^ (2) several pro-inflammatory or anti-inflammatory cytokines can accelerate the depletion and exhaustion of T cells with their respective functions. In addition, the virus may destroy secondary lymphoid tissues spleen and lymph nodes, leading to lymphopenia, which is supported by the observations of splenic atrophy, lymph node necrosis, and decreased lymphocyte numbers;^24,40–42^ (3) a nonhuman primate COVID-19 model showed that the impaired anti-viral T cell response may be attributed to the increased numbers of regulatory T cells (Tregs).^34^ However, we cannot exclude the possibility that depletion and exhaustion of lymphocytes resulted from anti-inflammatory therapies such as the administration of glucocorticoids.

Collectively, lymphopenia may represent a poor outcome of the illness. A retrospective, multicenter, emergency clinical trial in a Han Chinese population including 14,117 normal controls and 69 hospitalized COVID-19 patients (25 severe or critical and 44 mild) showed that lymphopenia occurred in almost 100% of the severe/critical cases, and the number of lymphocytes progressively decreased with the progression of the disease and deterioration of clinical status. The CD4^+^ and CD8^+^ T lymphocyte subsets showed a marked difference between mild and severe cases. Therefore, the authors suggested that analyzing the counts and percentages of lymphocytes at admission immediately contributes to improved clinical prognosis.^43^

### Antibody-dependent enhancement

B cells are considered protective in SARS-CoV-2 infection by producing neutralizing antibodies against the ACE2 receptor to prevent viral entry.^44^ However, B cell-produced neutralizing antibodies are not necessarily protective, depending on the virus element it targets and also the Fc region of the antibody. ADE is a phenomenon wherein pre-existing neutralizing antibodies targeting one serotype of a given virus enhance the entry of not only that virus but also another serotype of the virus into leukocytes through the interaction between the Fc regions of the antibody and Fc receptors or complement receptors on such cells.^45–49^ The ADE phenomenon has been found in various viral infections such as dengue, *Flavivirus*, SARS-CoV, MERS-CoV, and Ebola.^45,48,50–52^

Recent studies have shown that relatively high levels of B cells or antibodies are positively associated with COVID-19 severity,^32,53,54^ implying the potential involvement of ADE in SARS-CoV-2 infection. In addition, a study has reported that the monoclonal antibody MW05 targeting SARS-CoV-2 may also induce ADE activity by binding to FcγRIIB receptors on the target cells in vitro. However, administration of an engineered antibody with Fc region mutation in vivo effectively protected animals from SARS-CoV-2 infection.^55^ This highlights the importance of developing protective neutralizing antibodies against SARS-CoV-2. Nevertheless, further investigations about ADE in SARS-CoV-2 infection are required to facilitate the development of vaccine- or antibody-based therapy for COVID-19.

### Neutrophilia

An increase in neutrophil count in COVID-19 patients is widely recognized. It is well known that under normal conditions, neutrophils play a protective role against infections by producing neutrophil extracellular traps (NETs) to kill extracellular pathogens;^56^ however, excessive neutrophil activation can also damage the surrounding cells and dissolve connective tissues.^57^

An earlier clinical trial including 138 patients from Wuhan, China, showed that neutrophil counts were increased in non-survivors compared to survivors and continued to increase until death in the non-survivors.^11^ Another clinical study that integrated transcriptomic, proteomic, and metabolomic platforms showed that neutrophil counts were increased in patients with severe, but not mild, COVID-19 as compared to healthy controls, and molecules associated with NETs were significantly upregulated in severe COVID-19 cases.^58^

The increased neutrophils manifested as both increased numbers of mature and immature cells. In a clinical trial that integrated single-cell RNA-sequencing with single-cell proteomics of blood and peripheral blood mononuclear cells (PBMCs), immature neutrophil precursors, and dysfunctional mature neutrophils expressing programmed death-ligand 1 appeared in severe COVID-19 cases.^59^ In addition, a single-cell sequencing analysis by Wilk et al.^32^ and a flow cytometry analysis by Ronit et al.^33^ also identified the appearance of neutrophil progenitors at various developmental stages in PBMCs or bronchoalveolar lavage fluid (BALF) of COVID-19 patients with ARDS.

Although the mechanism by which the virus promotes neutrophil development in COVID-19 is still poorly understood, McElvaney et al.^60^ found that the levels of pyruvate kinase M2 (PKM2), a regulator of glycolysis^61^ and coactivator of hypoxia-inducible factor-1α,^62^ as well as phosphorylated PKM2 were higher in the neutrophils of COVID-19 patients in the ICU than in those of non-ICU COVID-19 patients. This indicates that neutrophils undergo immunometabolic reprogramming in severe COVID-19 cases, which represents a potential intervention target for excessive neutrophil generation and activation in severe or critical COVID-19.

### Dysregulation of monocytes and macrophages

Monocytes and macrophages are the major innate immune cells in infection and inflammation not just by virtue of their higher numbers but also by their functions. A single-cell RNA-sequencing analysis showed that classic CD14^+^ monocytes were significantly increased, whereas nonclassical CD16^+^ monocytes and intermediate CD14^+^CD16^+^ monocytes were remarkably reduced in the blood of COVID-19 patients with severe symptoms. Classical monocytes can differentiate into macrophages in tissue to initiate an inflammatory response, whereas nonclassical monocytes were viewed as anti-inflammatory as they can maintain vascular homeostasis,^63^ which may explain the phenotypic shift of circulating monocytes from CD16^+^ to CD14^+^. Analysis of the differentiation profiles of BALF and circulating monocyte–macrophages from the same patient revealed a transition course of blood-toward-BALF. More importantly, multiple pro-inflammatory cytokines and chemokines were highly expressed by the BALF monocyte–macrophages, suggesting that the cells are inflamed.^64^ Another single-cell sequencing analysis of peripheral blood samples also showed that CD16^+^ monocytes were remarkably depleted in COVID-19 patients with ARDS, with a phenotypic shift from CD16^+^ to CD14^+^. However, significant upregulation of genes encoding pro-inflammatory cytokines or chemokines were not found in peripheral monocytes, indicating that peripheral monocytes may not be responsible for the progression of CS in COVID-19.^32^ Moreover, phenotyping leukocyte subpopulations in BALF and blood of COVID-19 patients with ARDS showed that the expression of activation markers such as CD16, CD64, CD69, and HLA-DR was higher in BALF macrophages than in peripheral macrophages.^33^ Collectively, these existing studies were generally consistent and revealed the course of blood-toward-BALF transition and the contribution of pulmonary monocyte–macrophages to CS via the release of multiple pro-inflammatory cytokines and chemokines during severe COVID-19.

Interestingly, a two-cohort study showed that activated HLA-DR^high^CD11c^high^CD14^+^ monocytes were increased in the PBMCs of patients with mild COVID-19, whereas dysfunctional HLA-DR^low^CD163^high^ (indicative of anti-inflammatory function) CD14^+^ monocytes were observed in severe COVID-19 cases.^59^ This merits further investigation to understand the underlying mechanism and clinical significance.

### Reduced or delayed IFN-I response

**Fig. 3:**
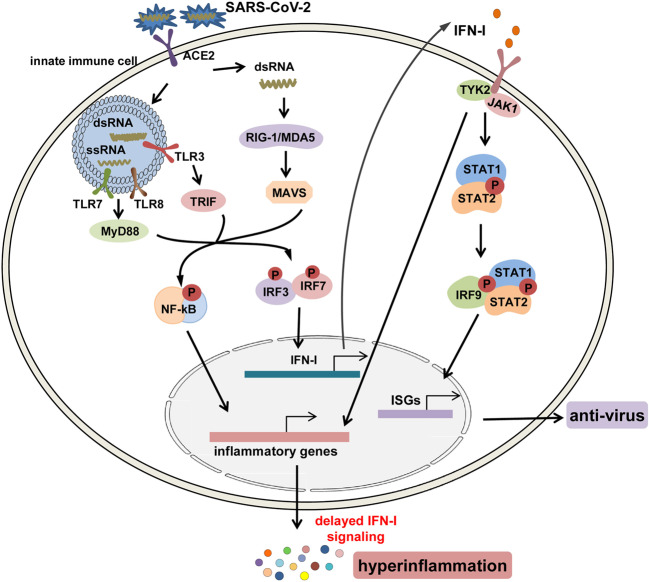
The signaling pathways for the production and function of IFN-I after SARS-CoV-2 infection. After infection, the genomic ssRNAs and replicative dsRNA intermediates of SARS-CoV-2 are recognized by endosomal toll-like receptors TLR3, 7, 8, and cytosolic RNA sensors, RIG-1/MDA5; next, downstream transcription factors including NF-κB and IRF3/7 are activated to induce the production of pro-inflammatory cytokines and IFN-I. IFN-I can activate the JAK1/TYK2–STAT1/2 pathway, promoting the formation of the STAT1/2/IRF9 complex and initiating the transcription of ISGs to produce anti-virus mediators, and it can also nonconventionally activate inflammatory pathways such as NF-κB and MAPK pathways to induce the expression of pro-inflammatory cytokines and paradoxical hyperinflammation in COVID-19

The IFN-I response is the first line of protective response and critical to combat viral infections by promoting viral clearance and regulating innate and adaptive immune responses.^65^ Although the detailed mechanism is still unknown when the infection occurs, the RNA of SARS-CoV-2 virus may be recognized by innate immune cells via pattern recognition receptors (PRRs) including toll-like receptor (TLR); retinoic acid-inducible gene-I (RIG-I)-like receptors (RLRs)/melanoma differentiation-associated gene 5 (MDA5); and NOD-like receptors (NLRs).^66^ Subsequently, downstream transcription factors including nuclear factor-κB (NF-κB), activator protein-1 (AP-1), and IFN regulatory factor 3/7 (IRF3/7) are activated to promote the transcription of pro-inflammatory cytokines and IFN-I. The IFN-I can activate the Janus kinase 1 (JAK1)/tyrosine kinase 2–signal transducer and activator of transcription 1/2 (STAT1/2) pathway, promoting the formation of the STAT1/2/IRF9 complex and initiating transcription of IFN-stimulated genes (ISGs) (Fig. 3).^66,67^

However, accumulating evidence has suggested that the protective IFN-I response was remarkably reduced in severe COVID-19 patients.^26,68,69^ At least two mechanisms have been proposed to explain the deficient IFN-I response: (1) previous studies have suggested that SARS-CoV employs various mechanisms to inhibit IFN response, especially through the components of its structural proteins such as M protein,^70^ N protein,^71^ open-reading frame 3a (ORF3a) protein,^72^ and ORF6 protein.^73^ Considering that the structure of SARS-CoV-2 is similar to that of SARS-CoV, it can be speculated that SARS-CoV-2 may exert similar effects on IFN response. For example, Yang et al.^74^ found that the NSP1 protein of SARS-CoV-2 can inhibit STAT1 phosphorylation and ISG transcription. (2) Decreased plasmacytoid dendritic cells (pDCs) may be partly responsible for the deficient IFN-I response. Sufficient evidence has suggested that pDC is a prominent producer of IFN-I upon viral infection.^75–77^ However, existing studies showed that counts of pDCs were decreased in the blood of COVID-19 patients, especially in severe cases.^26,32^

Of note, although the levels of systemic IFN-I were low, local IFN-I and ISGs were noticeable in the BALF of some critically ill patients,^78^ which are related to the phenomenon of delayed IFN-I response.^69^ Evidence has confirmed that a delayed IFN-I response not only impedes viral clearance but also induces paradoxical hyperinflammation, thereby aggravating the immunopathological response.^79,80^ Collectively, these studies suggest that IFN-I-based therapy for COVID-19 should be applied as early as possible after the infection is confirmed.

### Cytokine storm

In this section, we systemically review and discuss the characteristics, possible induction mechanism, pathogenesis, and diagnosis of CS in COVID-19.

Sufficient evidence has revealed the components and characteristics of CS in the patients with severe COVID-19, which are composed of an array of cytokines including interleukin-1 (IL-1), 2, 6, 7, 8, 10, 12, 17, 18; tumor necrosis factor-α (TNF-α); IFN-γ; granulocyte colony-stimulating factor (G-CSF); granulocyte–macrophage colony-stimulating factor (GM-CSF); and monocyte chemoattractant protein-1 (MCP-1).^26,33,60,81–86^ Reports of hemophagocytosis and clinical benefits from cytokine-targeted therapies in severe COVID-19 patients further confirmed the existence and pathogenesis of COVID-CS.^87,88^ Existing evidence has characterized and distinguished COVID-CS from CS in variable conditions such as HLH induced by specific viral infections,^89^ macrophage activation syndrome (MAS) occurring after autoimmune disorders,^90^ and cytokine release syndrome (CRS) caused by CAR T cell therapy^23^ in several aspects. First, COVID-CS involves more inflammatory cytokines than other CS conditions, thereby providing an explanation for the aggressive nature of COVID-19. Second, lymphopenia, although relatively less frequent in other CS, was often observed in patients with COVID-CS,^88^ suggesting that COVID-CS may be mainly attributed to innate—rather than adaptive immune cells. Finally, compared with bacterial infection-induced CS (e.g., sepsis), the treatment of COVID-CS is more challenging, because blocking inflammatory cytokine function without effective anti-viral drug support may exacerbate the infection.

The initiation of COVID-CS induction during infection and the predominant causative cytokine in COVID-19 immunopathology remain largely unknown. Despite the lack of definite pathogen-associated molecule pattern (PAMP) of SARS-CoV-2, in analogy with SARS-CoV and MERS-CoV, it can be speculated that upon cellular entry of SARS-CoV-2 via its ACE2 receptor, viral genomic single-stranded RNA or other RNA compositions (double-stranded RNA) as PAMPs can be sensed by the related PRRs, including TLRs and RLRs in host cells. The downstream transcription factors IRF3/7 and NF-κB are activated following PAMP recognition to induce the production of IFN-I and pro-inflammatory cytokines, respectively.^91–93^ However, as mentioned above, the protective IFN-I response is quickly and selectively abrogated by SARS-CoV-2 via different mechanisms. This is accompanied by an overwhelming production of pro-inflammatory cytokines in the context of COVID-19, which not only impairs viral clearance but also promotes paradoxical hyperinflammation including CS. Therefore, from the immunology perspective, COVID-CS may be an unfortunate event whereby the intended host immune response combating the SARS-CoV-2 has lost control and transformed into an inflammatory type.^15^

**Fig. 4:**
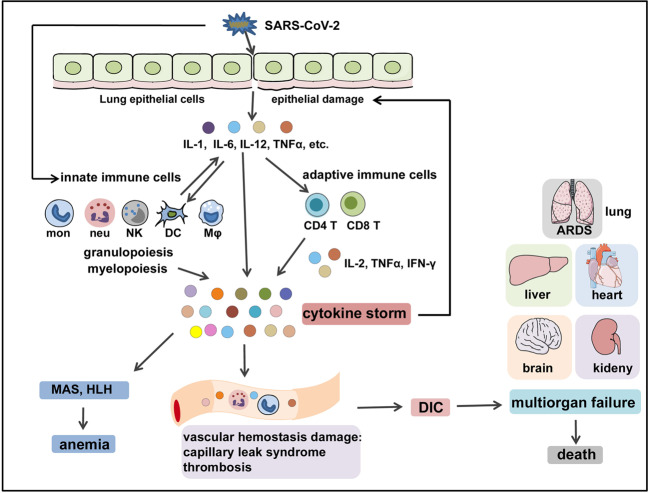
The immunopathological mechanisms of COVID-CS. SARS-CoV-2 infects the epithelial cells or immune cells, causing tissue damage and release of inflammatory cytokines (e.g., IL-1, IL-6, IL-12, and TNFα) by epithelial cells and immune cells. These inflammatory cytokines then recruit innate immune cells (monocytes, macrophages, neutrophils, DCs, and NK cells) and activate adaptive immune cells (CD4^+^ T cells and CD8^+^ T cells) to induce the occurrence of myelopoiesis and emergency granulopoiesis, as well as the production of sustained and excessive circulating cytokines that can further aggravate epithelial damage. In addition, overproduction of systemic cytokines triggers macrophage activation (i.e., MAS) and erythro-phagocytosis (i.e., HLH), resulting in anemia and gives rise to perturbation of vascular hemostasis, resulting in capillary leak syndrome, thrombosis, and DIC. These events together lead to ARDS, multiorgan failure, and death. HLH hemophagocytic lymphohistiocytosis, MAS macrophage activation syndrome, ARDS acute respiratory distress syndrome, DIC disseminated intravascular coagulation

In SARS-CoV-2 infection, the virus infects the respiratory epithelial tissue and activates local innate immune cells to release inflammatory cytokines such as IL-1, IL-6, IL-8, IL-12, TNF-α, and other chemokines. These inflammatory cytokines and chemokines then recruit more innate immune cells (monocytes, macrophages, neutrophils, DCs, and NK cells) and activate adaptive immune cells (CD4^+^ and CD8^+^ T cells) from the peripheral tissues to produce sustained inflammatory cytokines like IL-2, IFN-γ, and TNF-α, which induce myelopoiesis and emergency granulopoiesis that further aggravate lung and epithelial damage (Fig. 4). In addition, overproduction of systemic cytokines, particularly IL-2, IFN-γ, GM-CSF, and TNF-α, triggers macrophage activation (i.e., MAS) and erythro-phagocytosis (i.e., HLH), resulting in anemia,^94,95^ as well as causes perturbation of coagulation and vascular hemostasis, resulting in capillary leak syndrome, thrombosis,^96^ and DIC. These events together lead to ARDS, multiorgan failure, and death (Fig. 4).^15^ Of note, the host immunoregulatory system is usually capable of retaining and fine-tuning the protective inflammation to an appropriate level. Regulatory cells such as Tregs^97,98^ can produce regulatory cytokines like IL-10 and tumor growth factor-β to antagonize overactivated immune responses.^99,100^ However, aggressive inflammatory conditions such as CS cannot be calmed by the regulatory system’s natural ability.

Although the immunological and pathological understanding of COVID-CS has provided valuable information regarding the development of diagnosis and treatment strategies, detailed guidelines are still lacking. Developing scoring systems such as HScore, MS score, HLH-2004, Penn grading scale, and the Common Terminology Criteria for Adverse Events may be beneficial to predict COVID-CS or related outcomes. Caricchio et al.^101^ proposed predictive criteria for COVID-CS diagnosis. These criteria comprise three clusters: (1) albumin <2.87 mg/mL, lymphocytes <10.2%, neutrophil absolute count >11.4 × 10^3^/mL; (2) alanine aminotransferase >60 IU/L, aspartate aminotransferase >87 IU/L, d-dimer >4930 ng/mL, lactate dehydrogenase >416 U/L, troponin I >1.09 ng/mL; and (3) anion gap <6.8 mmol/L, chloride >106 mmol/L, potassium >4.9 mmol/L, and blood urea nitrogen:creatinine ratio >29. In addition, ferritin >250 ng/mL and C-reactive protein (CRP) >4.6 mg/dL are added for the reassurance of ongoing systemic inflammation. In another study, the authors proposed that a diagnostic criterion including peripheral blood oxygen saturation to the fraction of inspired oxygen (SpO_2_/FiO_2_), CRP, ferritin, cytokines/chemokines, and neutrophil/lymphocyte ratio may have a strong diagnostic power for COVID-CS.^102^ Mehta et al.^13^ proposed that prospective screening for hyperinflammation using laboratory assays and the HScore should be performed in all severely ill COVID-19 patients to identify COVID-CS. Despite the requirement for further validation, these criteria indeed provide constructive suggestions for the development of officially recognized guidelines for COVID-CS.

**Fig. 5:**
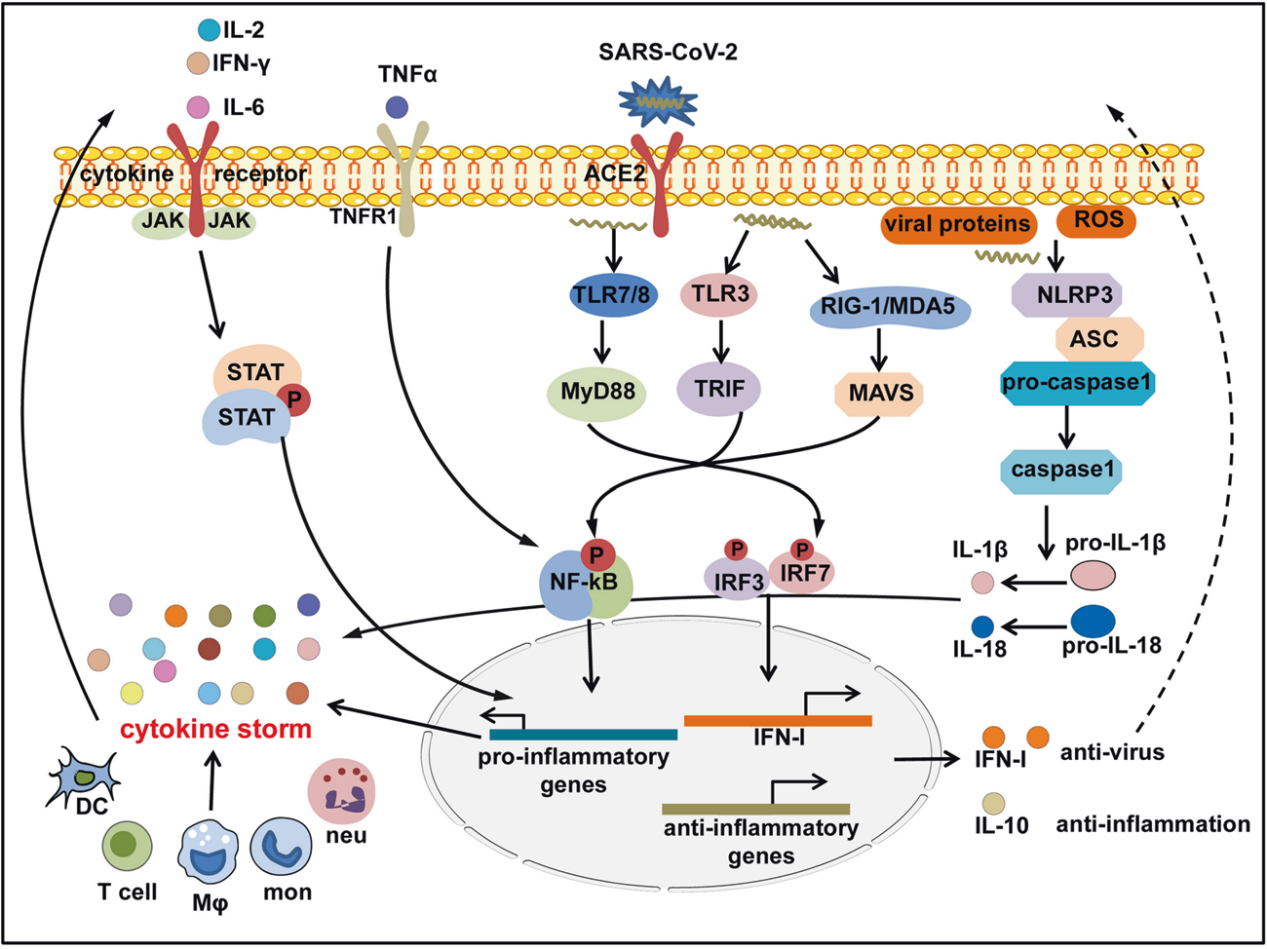
Inflammatory signaling cascades activated in COVID-CS. SARS-CoV-2 enters the host cells and is sensed by toll-like receptors (TLRs)3, 7, 8; RIG-I-like receptor, RIG-I or MDA5; and NOD-like receptor, NLRP3, that can also be directly activated by viral proteins or ROS released by apoptotic or inflamed cells. The downstream transcription factors IRF3/7 are activated to induce the production of IFN-I and related paradoxical hyperinflammation; NF-κB is activated to induce the production of pro-inflammatory cytokines; and NLRP3 inflammasome is activated to induce the production of mature IL-1β and IL-18. Pro-inflammatory cytokines such as IL-6, IL-2, TNF-α, and IFN-γ in turn activate the JAK-STAT or NF-κB signaling via binding to their receptors expressed on immune cells to induce more production of pro-inflammatory genes, forming a positive feedback to trigger the threshold of CS. Conversely, regulatory cytokines like IL-10 are compensatorily produced to antagonize immune hyperactivity

COVID-CS is a complicated and dynamic inflammatory process caused by a group of cytokines from initiation, immune cell hyperactivation, to organ dysfunction. The development of precise therapeutic intervention in appropriate time is required to effectively control COVID-CS. In principle, the treatment strategy is to control ongoing inflammatory response by specifically or nonspecifically targeting inflammatory cytokines or related signaling pathways and to resume the host immunoregulatory system. Herein, we discuss the role of the key cytokines and associated signal pathways involved in COVID-CS (Fig. 5).

#### IL-6/JAK/STAT signaling

A retrospective, multicenter study including 150 patients from Wuhan, China showed significantly elevated levels of IL-6 in severe COVID-19 cases.^103^ A study from Germany showed that IL-6 >80 pg/mL in combination with CRP >97 mg/L presented a relatively high specificity and sensitivity to predict respiratory failure.^104^ In addition, other studies have also shown the remarkably increased serum levels of IL-6 in patients with severe COVID-19.^68,105,106^

IL-6, first produced by monocytes, macrophages, and DCs, serves as a prominent activator of the JAK/STAT3 pathway in the context of inflammation. Recent studies have determined that the IL-6–JAK–STAT3 axis is closely related to the severity of COVID-19,^107,108^ and the levels of phosphorylated STAT3 were higher in different subsets of leukocytes in COVID-19 patients than in healthy controls.^109^ IL-6 deploys two signaling pathways—classic *cis*-signaling and *trans*-signaling—to trigger the activation of downstream JAK/STAT3 signaling through the membrane-bound (mIL-6R) and soluble form of IL-6R (sIL-6R), respectively.^110^ In *cis*-signaling, IL-6 binds to mIL-6Rs that are restrictedly expressed on immune cells, forming an IL-6/IL-6R/gp130 complex to activate downstream JAK/STAT3, Akt/mTOR, and MAPK signaling. This exerts pleiotropic effects on immune cells, which are manifested as promoted differentiation of T-helper type 17 (Th17), CD8^+^ T, and B cells; increased migration of neutrophils; and reduced development of Tregs.^111,112^ These, in turn, induce increased secretion of IL-6 and aggravate inflammation. In *trans*-signaling, circulating IL-6 binds to sIL-6Rs to form a complex, then binds with the gp130 dimer that is expressed on almost all cell types. The resultant activation of the JAK–STAT3 signaling occurs in cells with absent expression of mIL-6R, such as endothelial cells and vascular smooth muscle cells (VSMCs). The overwhelming activation of the IL-6–IL-6R–JAK–STAT3 pathway triggers the secretion of various mediators, such as IL-6 itself, IL-8, MCP-1, and vascular endothelial growth factor (VEGF), and reduces the expression of E-cadherin expressed on endothelial cells.^113^ Several studies have shown that MCP-1 can facilitate the formation of atherogenesis,^114^ production of adhesion molecules,^115,116^ and proliferation and migration of VSMCs,^117^ which partly explains the occurrence of cardiovascular symptoms observed in COVID-CS. In addition, increased VEGF and decreased E-cadherin can lead to vascular permeability and leakage and accelerate the presence of hypotension and pulmonary dysfunction in COVID-CS.^87^ Moreover, IL-6 can promote the secretion of various acute-phase proteins such as CRP, hepcidin, fibrinogen, thrombopoietin, complement C3, and ferritin in hepatocytes.^118,119^ Collectively, IL-6 contributes to both immune cell hyperactivation and target organ dysfunction in CS.

Evidence has suggested that the production of IL-6 can be induced by angiotensin II in inflamed vessels. The underlying mechanism is that angiotensin II binds to angiotensin II type 1 (AT1) receptor and then activates JAK/STAT signaling to induce IL-6 production.^120,121^ Interestingly, existing studies have shown that SARS-CoV may promote the expression of angiotensin II by downregulating ACE2,^122,123^ which potentially leads to the possibility that SARS-CoV-2 enhances IL-6 production via the angiotensin II/AT1 receptor/JAK/STAT axis, and the positive pro-inflammatory feedback of IL-6/JAK/STAT ultimately drives clinical signatures of COVID-19, especially COVID-CS.^124^

#### IFN-γ/JAK/STAT signaling

IFN-γ, mainly produced by macrophages, T cells, and NK cells, participates in immunological processes such as inflammation. It is a dominating driver of macrophage, NK, and T cell activation, and exerts a predominant effect on protective immunity against bacterial and viral infections through the activation of JAK1/JAK2 complex and downstream STAT1-IFN-γ-activated site (GAS) cascades.^125–127^

Evidence has shown that IFN-γ is highly involved in various CS-related disorders,^128^ as illustrated by its pathological role in primary HLH, a syndrome of failure to eliminate pathogens owing to impaired NK cell activity. In such cases, despite excessive T cell activation and large quantities of IFN-γ production, the increased IFN-γ levels fail to combat pathogens and instead cause immunopathology because of defective NK activity.^129^ Whether IFN-γ plays a specific role in COVID-CS is still unknown; however, considering its role in promoting immune cell activation, we can speculate that it contributes substantially to COVID-CS.

Numerous studies have reported elevated levels of IFN-γ in patients with COVID-19.^9,130,131^ Of note, one study showed that IFN-γ produced by CD4^+^ T cells was decreased in patients with severe disease compared to those with moderate disease, which could be explained by the reduced numbers and functional exhaustion of T cells in severe COVID-19,^132^ as described– above. Therefore, this study suggested that elevated levels of IFN-γ in COVID-19 are produced mainly by macrophages, not T cells.

#### TNFα/NF-κB signaling

TNFα is a well-known pro-inflammatory cytokine and is closely associated with many infectious, autoimmune diseases, and cancer, and is primarily produced by monocytes, macrophages, and T cells.^133,134^ NF-κB plays an evolutionarily conserved role in the immune system,^135,136^ especially in regulating the expression of various vital cytokines involved in inflammation.^137^ TNFα, as an initial driver of NF-κB activation, can activate the NF-κB signaling pathway to induce the expression of several pro-inflammatory and antiapoptotic genes through its receptor TNFR1 and a series of intermediate adapters.^138–140^ In turn, NF-κB can induce TNFα expression in the context of inflammation, such as lipopolysaccharide (LPS) stimulation.^141^ Therefore, TNFα/NF-κB signaling may play pathological roles in the stage of initiation and immune cell hyperactivation in CS by inducing apoptosis of epithelial cells to drive the epithelium–immune cell interplay and augmenting systemic inflammation.

Previous studies have found that excessive TNFα represented a poor prognosis in SARS-CoV and MERS cases,^142–145^ and inhibition of NF-κB improved pulmonary symptoms in SARS-CoV-infected mice.^146^ However, the role of TNFα in COVID-19 is, so far, still not entirely clear. Recent studies have reported elevated serum levels of TNFα in severe COVID-19 cases.^9,13,24,81^ In addition, a clinical trial from Wuhan, China, including 522 patients and 40 healthy controls showed that the concentration of TNFα was negatively correlated with T cell counts in COVID-19 patients.^27^ In contrast, a clinical trial from Chongqing, China, including 102 mild and 21 severe cases showed that TNFα levels were within the normal values in almost all COVID-19 patients (121/123).^147^ Thus, further research is urgently required to better understand the role of TNFα in COVID-CS. Although a recent report suggested that inhibition of the TNFα–NF-κB pathway may have protective effects in COVID-19,^124^ caution should be applied based on two aspects: (1) as mentioned above, the roles of TNFα in COVID-CS is still undefined; (2) blocking NF-κB nonspecifically may simultaneously impair its protective functions in cellular homeostasis, as exemplified by a general suppression on innate immunity.^148^

#### NLRP3/IL-1β signaling

IL-1β is perhaps the most well-studied member of the IL-1 family because of its prominent role in autoinflammatory diseases such as gout and chronic inflammatory arthritis.^149–151^ IL-1β is mainly secreted by macrophages via apoptosis and pyroptosis and exerts positive effects on the migration of immune cells to inflamed tissues; Th17 cell differentiation; expression and release of various cytokines and adhesion factors; and NF-κB pathway activation to form a positive feedback for its own production.^152,153^ Upstream, the NLRP3 protein forms a complex with apoptosis-associated speck-like protein containing a caspase recruitment domain (ASC) and cysteinyl aspartate-specific proteinase-1 (caspase-1), termed the NLRP3 inflammasome, to cleave the inactive IL-1β precursor to the mature form of IL-1β.^154,155^ Considering the positive roles of IL-1β in activating initiative and sustained inflammation,^156^ it was postulated that NLRP3-IL-1β signaling might be involved in COVID-CS.

Multiple indicators from previous evidence have suggested that IL-1β may contribute to CS in coronavirus infections.^157–159^ Zhang et al.^160^ reported the elevated levels of multiple cytokines including IL-1β in COVID-19 cases with severe symptoms, which were also associated with SARS, hypercoagulation, and DIC. Consistently, Huang et al.^9^ also showed high serum concentrations of IL-1β in COVID-19 patients. Moreover, a previous study showed that NLRP3 can be directly activated by viral proteins of SARS-CoV such as ORF3a and ORF8b,^161,162^ which were also found on the genome of SARS-CoV-2,^163^ suggesting a potentially similar effect of direct activation of the NLRP3 by SARS-CoV-2 protein. The potential roles of NLRP3 inflammasome in severe COVID-19 have been discussed in relevant reviews.^164,165^ Reactive oxygen species (ROS) was reportedly an initiator of NLRP3 activation.^166–168^ Thus, it was proposed that excessive production of ROS resulting from inflammation infiltration in severe COVID-19 may lead to NLRP3 activation and IL-1β precursor cleavage, further aggravating inflammation in COVID-CS.

#### IL-2/IL-2R/JAK/STAT5 signaling

IL-2 is mainly secreted by CD4^+^ T cells and plays crucial roles in the expansion and differentiation of CD4^+^ T, CD8^+^ T, NK, and other cells through the IL-2R–JAK–STAT5 signaling pathway.^169,170^ IL-2 can fine-tune immune responses and maintain self-tolerance,^171^ and its deficiency accounts for the occurrence of autoimmune diseases.^172^

Elevated levels of IL-2 have been reported in other types of coronavirus infections.^173,174^ Recent studies have also shown that the concentrations of IL-2 or IL-2R were elevated in COVID-19 patients, especially in those with severe illness.^9,13,24,132^ However, a clinical trial including 54 COVID-19 patients from Beijing, China, reported a contradictory result. The authors found that compared with severe patients (*n* = 14), those with a critical illness (*n* = 6) had remarkably reduced plasma levels of IL-2. Accordingly, IL-2Rα levels were significantly decreased in the PBMCs of patients with a severe and critical illness compared to common patients (*n* = 34) or healthy controls. Furthermore, the levels of JAK1 and STAT5 were significantly lower in all three groups than in normal controls. Considering the supportive roles of IL-2 in the expansion and differentiation of T cells, the authors speculated that the presence of lymphopenia especially in severe COVID-19 may be attributed to reduced levels of IL-2, IL-2R, JAK1, and STAT5.^175^ In additionally, CD4^+^ T cells are the primary source of IL-2, and the reduced numbers of lymphocytes during the stage of CS after COVID-19 infection can at least partly explain the reduced IL-2 level and downregulation of IL-2 signaling. Future investigations are required to confirm these findings in more patients.

#### IL-7/IL-7R signaling

IL-7/IL-7R signaling is essential for peripheral homeostasis and the survival, differentiation, and maintenance of T cells including CD4^+^ T, CD8^+^ T, naive T, and memory T cells.^176–179^ It is also indispensable for the development and maintenance of innate lymphoid cells (ILCs), formation of lymphoid structures, and barrier defense.^180^

Recent reports have shown elevated levels of IL-7 in COVID-19 patients, and these increases were related to disease severity.^9,13,147^ However, the impact of enhanced levels of IL-7 in COVID-19 is largely unknown. Considering the protective role of IL-7, we can speculate that the increase of IL-7 may be a feedback mechanism in response to the lymphopenia in patients with severe/critical COVID-19.

#### IL-10 signaling

IL-10 is an important immunoregulatory cytokine produced by a variety of immune cells including Th2 cells, Tregs, CD8^+^ T cells, B cells, DCs, macrophages, and NK cells, and signals through the IL-10R/JAK/STAT3 pathway. IL-10 exerts anti-inflammatory functions by directly limiting the innate immune-related functions of macrophages and DCs in an autocrine and paracrine manner or indirectly via improving Treg development. In addition, IL-10 can activate mast cells and strengthen the functions of CD8^+^ T, B, and NK cells.^181^

A clinical trial including 102 COVID-19 patients and 45 controls from Wuhan, China, showed that the serum IL-10 levels of patients with a critical illness (*n* = 17) were significantly higher than those of patients with moderate (*n* = 42) and severe (*n* = 43) illness; further, the IL-10 levels were positively correlated with the concentrations of serum CRP, indicating the potential of IL-10 as an indicator of disease severity.^182^ Huang et al.^9^ also reported the significantly high plasma levels of IL-10 in COVID-19 patients admitted to the ICU compared to those who were not. In addition, a follow-up clinical trial including 71 COVID-19 patients (53 mild and 18 severe) from Beijing, China and 18 controls showed that the production of IL-10 in the early stage was significantly correlated with disease severity.^183^

It can be speculated that the excessive production of IL-10 is a negative feedback mechanism to antagonize the hyperactivity of the immune system. However, when faced with an overwhelming secretion of inflammatory mediators and activation of pro-inflammatory cells in COVID-CS, the fine-tune function of IL-10 is rather inadequate. Thus, administration of IL-10 has been recommended to treat ARDS in COVID-19.^184^ However, a recent report showed that IL-10 may be detrimental in the initiation phase of SARS-CoV-2 infection by promoting T cell exhaustion. The authors proposed that blocking IL-10 with a neutralizing antibody in the initiation stage of SARS-CoV-2 infection may be a promising therapeutic approach.^185^

#### IL-12 signaling

IL-12, mainly produced by DCs, macrophages, and B lymphocytes,^186^ is a multifunctional immunoregulatory factor that can promote proliferation of Th1 and Th17 cells; improve the cytotoxicity of NK cells; and induce expression of IFN-γ in Th1 cells, NK cells, DCs, and macrophages via a positive feedback mechanism.^187,188^ Thus, IL-12 plays an aggressive role in CS by augmenting the activation of various immune cells.

Existing studies have reported that viral infections induce the production of IL-12 to defend against infections.^189–191^ For instance, during influenza virus infection, IL-12 is endogenously produced to induce the secretion of IFN-γ from Th1 and NK cells, thereby inhibiting viral replication.^190^ A previous study reported elevated plasma levels of IL-12 in patients infected by SARS-CoV.^192^ However, Huang et al.^9^ recently found that the plasma levels of IL-12p70 showed no difference between COVID-19 patients and healthy adults. Therefore, further research with large-sized samples is urgently required to determine the alterations and functions of IL-12 in COVID-19, especially COVID-CS.

#### IL-17 signaling

IL-17 (primarily IL-17A) is produced by Th17, CD8^+^ T, and group 3 ILC (ILC3) and participates in many pro-inflammatory processes and autoimmune diseases.^193–195^ Targeting IL-17 is now regarded as a common strategy to reduce the burden of several autoimmune diseases such as psoriasis and psoriatic arthritis. Nevertheless, the functions of this inflammatory cytokine vary, from being protective against infections to having detrimental pro-inflammatory effects, depending on the tissue type and location (gut, lung, or skin) where it is being expressed and its triggering factors.^196^

Increased levels of IL-17 were previously reported in SARS-CoV- or MERS-CoV-infected patients.^197,198^ In addition, IL-17 can augment lung injury and decrease overall survival through recruitment of neutrophils; stimulate the expression of pro-inflammatory factors; and induce the expression of G-CSF to prevent apoptosis in both ARDS and an LPS-induced acute lung injury model.^199,200^ Similarly, evidence has suggested that IL-17 levels were elevated in COVID-19 patients, especially in those with a severe and critical illness.^201^ Asrani and Hassan^156^ showed that IL-17 plays crucial roles in the stages of immune cell hyperactivation and target organ dysfunction in COVID-CS by promoting the recruitment of neutrophils and producing symptoms such as fever, matrix damage, tissue remodeling, and inflammatory infiltration.

Despite multiple evidence suggesting the potential of IL-17 as an intervention target for COVID-CS, studies have shown that the levels of IL-17 were within normal ranges in 102 and 21 patients with mild and severe COVID-19, respectively.^147^ Therefore, more clinical trials and fundamental research are required for further clarification.

#### GM-CSF signaling

GM-CSF is produced by endothelial, epithelial, hematopoietic, and other cell types.^202^ Under physiological conditions, low levels of GM-CSF can regulate the homeostasis of alveolar macrophages to maintain their antimicrobial functions.^203,204^ Under hyperinflammatory conditions such as CS, GM-CSF drives emergency myelopoiesis and recruits myeloid cells to the inflammatory sites to perpetuate inflammatory reactions.^205^ Increased levels of GM-CSF have been observed in SARS,^206^ ARDS,^207^ and CRS.^208^ A recent study also reported elevated GM-CSF levels in both severe and mild COVID-19.^9^

Given the role of GM-CSF in maintaining antimicrobial functions of alveolar macrophages, administration of GM-CSF to patients with early-stage COVID-19 may strengthen the alveolar wall and enhance viral clearance.^209^ In contrast, the blockade of GM-CSF signaling may achieve clinical benefits in COVID-CS.

## Cytokine-based interventions

**Fig. 6:**
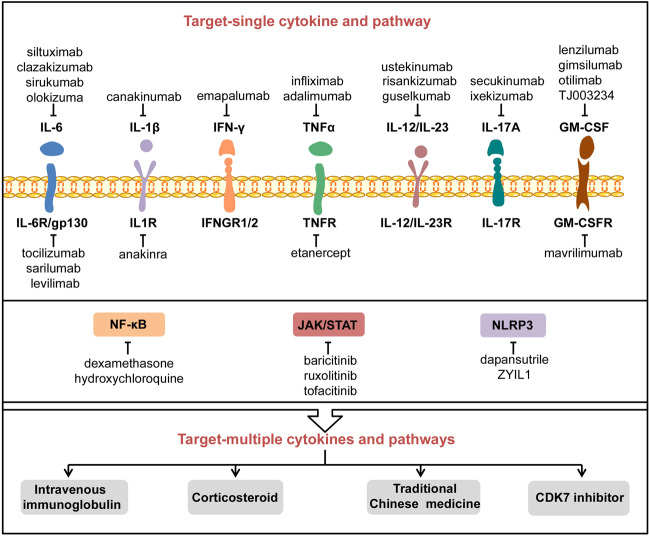
The potential inhibitors and therapies to counteract COVID-CS. A variety of inhibitors or drugs have been applied or are under consideration to treat COVID-CS, including those targeting a single pro-inflammatory cytokine or its receptor and related signal pathway. In addition, several treatments such as intravenous immunoglobulin, corticosteroids, traditional Chinese medicine, and CDK7 inhibitor may have the potential to counteract multiple cytokines and pathways involved in COVID-CS. CDK7 cyclin-dependent kinase 7

The current treatment for CS is mainly based on traditional anti-inflammatory drugs such as the administration of corticosteroids, chloroquine, and colchicines.^210,211^ Recently, biologics like recombinant cytokines; monoclonal antibodies against IL-6, IL-1β, TNF-α, and IFN-γ; and signaling pathway inhibitors are also available or in the pipeline for production. In this and the next section, we aim to discuss the key biologics that are currently or potentially applied to treat CS (Fig. 6).

### IFN-I

Administration of IFN-I has previously been investigated for the treatment of SARS-CoV and MERS-CoV. Generally, it was found to be relatively effective in vitro and in some animal models,^212^ although human studies were inconclusive.^213^

The research team at the University of Texas Medical Branch, USA, showed that low concentrations of IFN-α and IFN-β effectively reduced virus titer and inhibited viral replication in Vero cells in a dose-dependent manner, and they also found that SARS-CoV-2 is more sensitive than SARS-CoV to IFN-I in vitro.^214^ An uncontrolled, exploratory study from Tongji Medical College, Wuhan, China, showed that administration of IFN-α2b alone or in combination with arbidol remarkably accelerated virus clearance as well as the recovery of IL-6 and CRP to normal levels in COVID-19 patients.^215^ A randomized, multicenter, prospective, phase 2 clinical trial conducted at the University of Hong Kong showed that compared with the control group (lopinavir/ritonavir), COVID-19 patients in the triple group (IFNβ-1b plus lopinavir/ritonavir + ribavirin) exhibited significantly alleviated symptoms; shortened virus shedding and hospital stay; and improved inflammatory conditions (NCT04276688).^216^ A prospective study including 2944 healthy medical staff in the epidemic areas from Taihe Hospital in Shiyan City, Hubei, China, showed that the incidence rate of COVID-19 was zero in both the low-risk group (*n* = 2415) and the high-risk group (*n* = 529) after treatment with recombinant IFN-α nose drops for 30 days (NCT04320238),^217^ indicating the preventive roles of recombinant IFN-α in COVID-19. A trial comparing the clinical efficacy of subcutaneous IFN-β1a plus lopinavir/ritonavir with lopinavir/ritonavir alone; hydroxychloroquine; and remdesivir in COVID-19 is still ongoing (NCT04315948). Collectively, these studies suggested that IFN-I can inhibit SARS-CoV-2 infection and potential COVID-CS.

Another type of interferon—IFN-III—may also have a clinical benefit in COVID-19 treatment. Dinnon et al.^218^ confirmed the anti-viral effect of pegylated IFN-λ1a in both SARS-CoV-2-infected human airway epithelial cells and mouse models. Some studies have determined that MDA5 plays a leading role in activating IFN-I/III response to defend against SARS-CoV-2 in human epithelial cells,^219–221^ which may open a new avenue for enhancing IFN response in COVID-19. In addition, several clinical trials evaluating the clinical efficacy of IFN-λ are under investigation (NCT04343976, NCT04331899).

### IL-7

The supportive role of IL-7 in lymphocyte survival and expansion provides clinical implications for the recovery of function of lymphocytes in severe/critical COVID-19.

A case report from Hospices Civils de Lyon, France, showed that a period of recombinant human IL-7 (rhIL-7) treatment remarkably improved the immune function in a 74-year-old ICU patient with severe COVID-19, which manifested as elevated lymphocyte count and mHLA-DR expression. In this case, IL-7 was administered quite late, on day 24 of admission. Thus, the authors prospectively proposed that earlier administration of IL-7 may have indicated better clinical outcomes.^222^ In a case series from St. Luc University Hospital, Brussels, Belgium, the authors showed that patients in the IL-7 treatment group seemed to have higher levels of lymphocyte counts than those in the control group, without aggravated inflammation and pulmonary injury.^223^ Unfortunately, the absence of detailed phenotypic or functional studies on lymphocytes weakens the reliability of these studies. A multicenter, double-blind clinical trial in a UK-based cohort is currently evaluating whether the administration of CYT107 (a commercial product of rhIL-7) can result in clinical improvement of patients with severe COVID-19 through immune reconstitution (NCT04379076). Clark et al.^224^ proposed that administration of IL-7 in combination with dexamethasone may be an optimal treatment for severe COVID-19. The underlying mechanism is that dexamethasone enhances IL-7 activity by upregulating its receptor IL‐7Rα, which can also be used to explain the most effective properties of dexamethasone in the more severe stage of the disease.^225,226^

These studies indicate that appropriate administration of IL-7 alone or in combination with other agents should be considered as early as possible for critical COVID-19 patients with severe lymphopenia.

## Blockade of cytokines

### Blockade of IL-6

As mentioned above, IL-6 signaling is a leading inducer of COVID-CS. Currently, several drugs targeting IL-6 signaling such as IL-6 inhibitors (siltuximab, clazakizumab, sirukumab, olokizumab) and IL-6R inhibitors (tocilizumab, sarilumab, levilimab) are available.^227–229^

A retrospective clinical trial from Anhui, China, including 21 COVID-19 patients with severe or critical illness showed that five days of tocilizumab therapy immediately improved the clinical outcomes in most patients, manifested as decreased oxygen requirements, serum CRP concentrations, and hospital stays, as well as the rapid recovery of lymphocyte percentage.^230^ Another multicenter cohort study including 3924 COVID-19 patients in the ICU across 68 hospitals in the United States showed that the risk of mortality was lower in 433 patients who received tocilizumab treatment immediately after ICU admission than in those who did not receive early tocilizumab intervention.^231^ In addition, numerous reports evaluating the administration of tocilizumab for severe COVID-19 have also been published,^106,232–247^ and a total of 75 clinical trials are currently registered in ClinicalTrials. gov.

An open-label study from Italy including 56 patients with severe COVID-19 showed that after 28 days of follow-up, sarilumab treatment (*n* = 28) appeared to promote the recovery of patients with mild lung consolidation (<17%) at baseline.^248^ Another retrospective, single-center, clinical trial from Italy including 15 COVID-19 patients showed that sarilumab treatment improved the clinical symptoms and reduced serum CRP levels in most patients (*n* = 10).^249^ Currently, a total of 17 clinical trials on sarilumab are registered in ClinicalTrials.gov for the treatment of COVID-19.

A randomized, phase 3, clinical trial evaluating the administration of levilimab in patients with severe COVID-19 has been completed, but the results have not yet been published (NCT04397562).

An observational, controlled cohort study from Italy including 30 patients requiring ventilator support showed that the 30-day mortality was significantly reduced in those receiving siltuximab plus optimal supportive care compared with the control patients who received only optimal supportive care; ~50% of patients receiving siltuximab treatment were finally discharged (NCT04322188).^250^ Three other clinical trials evaluating the efficacy and safety of siltuximab in severe COVID-19 are underway (NCT04329650, NCT04330638, NCT04486521).

In general, published clinical trials showed positive results with respect to some common IL-6 or IL-6R antagonists for the treatment of COVID-CS. Several clinical trials of other IL-6 or IL-6R antagonists including clazakizumab (NCT04381052, NCT04494724, NCT04343989, NCT04363502, NCT04348500, NCT04659772); sirukumab (NCT04380961); and olokizumab (NCT04380519, NCT04452474) have been registered in ClinicalTrials.gov to investigate their therapeutic potential in COVID-CS.

### Blockade of IL-1β

Considering the pathological role of IL-1β signaling in CS, several drugs targeting IL-1β signaling, including IL-1β antagonist canakinumab and IL-1 receptor antagonist anakinra, may offer clinical benefits in the treatment of COVID-19, particularly in CS.

A retrospective, three-center, clinical trial from France including 22 patients with severe/critical COVID-19 showed that after >8 days of treatment, patients in the anakinra group (*n* = 12) presented improved clinical conditions as well as decreased mechanical ventilation requirements and serum CRP concentrations, as compared with the control group (*n* = 10) (INDS, MR4115050520).^251^ A prospective, cohort study from the Netherlands showed that several clinical parameters including temperature; white blood cell count; and levels of plasma ferritin, creatinine, procalcitonin, and bilirubin were decreased after 28 days of anakinra treatment.^252^ Several other studies have also investigated the clinical effects of anakinra on COVID-19.^253–261^ A total of 35 clinical trials are currently registered in ClinicalTrials.gov.

A retrospective study from Italy reported for the first time that subcutaneous administration of 300 mg canakinumab rapidly reduced systemic inflammation and improved oxygenation in COVID-19 patients (*n* = 10) who presented hyperinflammation but did not require mechanical ventilation.^262^ Another single-center, cohort study from Italy enrolled 34 non-ICU patients with mild or severe COVID-19, with 17 receiving standard treatment and 17 receiving 300 mg of subcutaneous canakinumab. The results showed that canakinumab treatment significantly increased the PaO_2_/FiO_2_ ratio and reduced inflammatory indices.^263^ These two studies suggest that canakinumab treatment may have therapeutic potentials in non-ICU patients with mild or severe COVID-19. Several other reports have also evaluated the administration of canakinumab in COVID-19,^264–266^ and six clinical trials are currently registered in ClinicalTrials.gov (NCT04348448, NCT04476706, NCT04362813, NCT04365153, NCT04510493, NCT04278404).

### Blockade of IFN-γ

Emapalumab, a monoclonal antibody targeting IFN-γ, has been approved to treat primary HLH, a condition with elevated serum levels of IFN-γ.^267^ Considering the contribution of IFN-γ to CS as mentioned above, emapalumab may be effective in the treatment of COVID-CS.

A randomized, multicenter, clinical trial from Italy was registered to investigate the efficacy of emapalumab treatment in combination with anakinra to alleviate hyperinflammation and improve respiratory conditions (NCT04324021). Unfortunately, this trial is now terminated and no further data are currently available. Therefore, other randomized, controlled clinical trials are urgently required to address these issues.

### Blockade of TNFα

Recent studies have provided the theoretical and practical bases to support TNFα blockade as a potential strategy for excessive cytokine release and hyperinflammation in COVID-19.^268–271^

Etanercept is a soluble TNFα receptor fusion protein that has been used to treat toxic epidermal necrolysis (TEN), a condition of systemic hyperinflammation. Owing to the similarities in clinical manifestations and pathological characteristics between COVID-19 and TEN, Chen et al.^268^ proposed the temporary use of etanercept as a valuable approach to treat severe COVID-19. A case report described that a 60-year-old man who received subcutaneous etanercept treatment for spondyloarthritis prior to SARS-CoV-2 infection presented no signs of respiratory failure and progressive deterioration and showed rapid recovery from COVID-19.^269^ Infliximab is another clinically approved TNFα blocker. A total of four clinical trials of infliximab evaluating its therapeutic potential in COVID-19 are currently underway (NCT04425538, NCT04734678, NCT04593940, NCT04344249). Adalimumab is a monoclonal antibody targeting TNFα and is currently undergoing evaluation in two clinical trials (ChiCTR2000030089, NCT04705844).

Despite these promising results, we cannot exclude the possibility of chance, and hence, randomized, controlled, prospective clinical trials with a large sample size are urgently required for further validation.

### Blockade of IL-12/IL-23

IL-12/23 inhibitors currently used in the clinic include risankizumab, guselkumab, tildrakizumab (targeting IL-23p19), and ustekinumab (targeting IL-12/IL-23p40) mainly for chronic inflammatory and autoimmune diseases such as psoriasis and inflammatory bowel disease.^272–276^ A recent review proposed IL-12/IL-23 or IL-23 inhibitors as potential interventional targets for the ongoing COVID-19 pandemic.^277^

Several case reports have described the clinical efficacy of IL-12/IL-23 inhibitors including ustekinumab, guselkumab, risankizumab, and risankizumab in COVID-19 patients with psoriasis.^278–282^ The reliability of case reports is relatively weak, which is why randomized, controlled, prospective clinical trials are so important.

A multicenter, randomized, clinical trial is ongoing to evaluate the efficacy of risankizumab alone or risankizumab in combination with remdesivir in COVID-19 (NCT04583956).

### Blockade of IL-17A

Several review articles have proposed targeting IL-17A signaling as an intervening measure for patients with COVID-CS.^196,283–289^

Case reports from Italy have shown that patients with a history of psoriasis and previous treatment with IL-17A antagonists including secukinumab and ixekizumab showed relatively mild COVID-19 symptoms or were even asymptomatic.^290–292^ A retrospective, observational, multicenter clinical trial from Italy containing 5206 patients with psoriasis who have been prescribed medications including IL-17 inhibitors showed that only four patients were hospitalized for COVID-19, and no deaths occurred,^293^ indicating the protective role of IL-17A inhibitors in COVID-CS and ARDS. However, using the general Italian population as the control group reduces the degree of standardization and reliability of this trial. The absence of standard clinical or experimental diagnosis for COVID-19 is another limitation. In addition, other prospective, randomized, clinical trials evaluating the administration of ixekizumab (NCT04724629) and secukinumab (NCT04403243) in COVID-19 are also underway. In addition, many researchers have proposed that simultaneously targeting IL-17A signaling and synergic IL-6 signaling may offer more clinical benefit for COVID-19 patients, particularly for those who experience CS.^283,286^

### Blockade of GM-CSF

Several studies have reported the protective roles of GM-CSF in the early stage of infection,^294–296^ and numerous clinical trials of human recombinant GM-CSF including sargramostim and molgramostim have been registered for the treatment of COVID-19; however, as mentioned above, GM-CSF indeed exerts a pathological function in the phase of CS, implying that blocking GM-CSF signaling may achieve clinical benefits in COVID-CS.

Mavrilimumab is a monoclonal antibody against GM-CSF-Rα.^297^ A prospective cohort study from Italy including 39 patients with severe COVID-19 showed that patients in the mavrilimumab group (*n* = 13) showed earlier improvement, lesser progression to mechanical ventilation, and faster fever resolution than those in the control group (*n* = 26).^298^ In addition, several clinical trials evaluating the administration of mavrilimumab in severe COVID-19 have been registered (NCT04447469, NCT04463004, NCT04492514, NCT04399980, NCT04397497).

Lenzilumab is a recombinant monoclonal antibody against human GM-CSF. A case–cohort study including 39 patients with severe COVID-19 from the USA reported that compared to the control group (*n* = 27), patients who received intravenous treatment with lenzilumab (*n* = 12) exhibited significantly rapid clinical improvement; reduced progression to ARDS; and decreased inflammatory markers and inflammatory myeloid cells.^299^ In addition, several clinical trials for lenzilumab have been registered for the treatment of severe COVID-19 (NCT04351152, NCT04583969, NCT04534725).

These clinical trials showed that blockade of GM-CSF signaling indeed improved clinical outcomes in patients with COVID-CS. Moreover, several other GM-CSF inhibitors such as gimsilumab, otilimab, and TJ003234 are undergoing clinical evaluation as potential COVID-19 therapy (NCT04351243, NCT04376684, NCT04341116, respectively).

## Blockade of signaling pathways

### Blockade of JAK/STAT signaling

The JAK/STAT pathway lies downstream of various cytokines involved in the CS. Thus, several studies have proposed that the JAK/STAT signaling inhibition may be a valuable preventive or therapeutic option for COVID-CS.^126,300–305^ The clinical efficacy of various JAK inhibitors (JAKinibs) such as tofacitinib targeting JAK1 and JAK3^306,307^ as well as baricitinib and ruxolitinib, both targeting JAK1 and JAK2,^308–310^ are currently under investigation in clinical trials in the context of COVID-19.

Hoang et al.^311^ found that baricitinib treatment significantly improved the inflammatory condition in SARS-CoV-2-infected rhesus macaque, as manifested by reduced inflammatory cell infiltration and neutrophil recruitment; limited lung pathology; and suppressed expression of pro-inflammatory mediators in lung macrophages. Baricitinib has also been evaluated in a series of clinical trials. An observational, longitudinal trial including 76 COVID-19 patients showed that compared with the control group (*n* = 56), patients in the baricitinib group (*n* = 20) presented remarkably reduced serum levels of IL-6, IL-1β, and TNF-α; accelerated recovery of blood T and B cell counts; increased production of antibodies against SARS-CoV-2; and progressively increased PaO_2_/FiO_2_ ratio (NCT04438629).^109^ Another observational cohort study from Spain showed that baricitinib improved lung function in patients with moderate-to-severe COVID-19 receiving corticosteroid treatment.^312^ Interestingly, existing studies showed that besides the acknowledged inhibitory effects on CS, baricitinib can also dampen ACE2-mediated SARS-CoV-2 endocytosis by inhibiting AP2-associated protein kinase 1 and cyclin G-associated kinase,^313,314^ which serves as another mechanism of its action in COVID-19. Moreover, several other reports have also been published,^313,315–322^ and numerous clinical trials of baricitinib are registered at ClinicalTrials.gov to evaluate its clinical effects in severe COVID-19.

Several studies have shown that ruxolitinib may also be effective in the treatment of severe/critical COVID-19.^323–326^ In a prospective, multicenter, single-blind, phase 2 clinical trial including 41 COVID-19 patients from Wuhan, China, compared with patients in the placebo group (*n* = 21), those in the ruxolitinib group (*n* = 20) exhibited remarkably reduced levels of seven cytokines, as well as a faster rate of clinical improvement and lymphocyte-count recovery.^325^ In addition, several other clinical trials for ruxolitinib evaluation and six clinical trials that are evaluating the administration of tofacitinib in COVID-19 patients have been registered at ClinicalTrial.gov (NCT04412252, NCT04415151, NCT04750317, NCT04469114, NCT04390061, NCT04332042).

Despite these promising clinical data, JAKinibs should be used with caution because of their side effects, based on the following considerations: (1) nonselective inhibition of the JAK/STAT pathway increases the risk of secondary infection such as herpes zoster virus reactivation given its general inhibitory effects on multiple aspects of physiological actions, including the innate immune system.^327,328^ In addition, considering the general immunosuppressive nature, some researchers are concerned that continuous treatment with JAKinibs for autoimmune diseases may increase the risk of SARS-CoV-2 infections or contribute to poor outcomes in COVID-19. Hence, several retrospective clinical trials have been conducted to address these concerns;^329–334^ (2) critical COVID-19 is commonly accompanied by coagulopathy and thrombosis, and the Food and Drug Administration has warned that administration of some JAKinibs has increased the risk of thrombosis.^335^ Thus, developing JAKinibs with better specificity could be a future direction of research aimed to prevent/reduce CS and improve the survival of COVID-19 patients.

### Blockade of NF-κB signaling

The overwhelming expression of multiple pro-inflammatory proteins in COVID-CS has indicated the central roles of pro-inflammatory signaling pathways, and in particular, the NF-κB pathway. Immunomodulation of NF-κB activation has been proven effective in SARS-CoV-infected cells or mice.^146^ Thus, recent reviews have proposed that the NF-κB pathway represents a potential therapeutic target for critical COVID-19 illness.^336–338^

An in vitro study showed that phillyrin (KD-1), a well-studied anti-inflammatory and antioxidative agent, significantly reduced the replication of SARS-CoV-2 and expression of pro-inflammatory factors in Huh-7 cells via inhibition of the NF-κB signaling pathway.^339^ Another study showed that a novel pyrazole derivative remarkably reduced the expression of IL-6, TNFα, and IL-1β in LPS-stimulated RAW267.4 cells by inhibiting NF-κB signaling pathway activation.^340^ It has been suggested that blocking phosphorylation of the inhibitor of NF-κB kinase subunit beta, a primary downstream protein of NF-κB signaling, with pharmacological inhibitors may be an effective approach for COVID-CS treatment.^339^ Moreover, Liu Shen capsules, a traditional Chinese medicine, were also reported to exert anti-viral and anti-inflammatory effects in SARS-CoV-2-infected Huh-7 and Vero E6 cells, respectively, by suppressing the NF-κB signaling cascade.^341^ The effect of several anti-inflammatory or anti-viral drugs on COVID-19 such as dexamethasone,^342^ hydroxychloroquine,^343^ macrolide antibiotics,^344,345^ and *N*-acetylcysteine^346,347^ are also related to NF-κB cascade inhibition.

Despite the existence of various nonselective agents for NF-κB inhibition, developing selective NF-κB inhibitors and a series of clinical trials are urgently required to further validate the clinical benefits.

### Blockade of NLRP3 signaling

Several studies have shown that NLRP3 inflammasome is a potential therapeutic target for COVID-CS.^164,348,349^

NLRP3 signaling inhibition may be a potential mechanism of action for several anti-inflammatory drugs effective in COVID-19, such as colchicine. Studies have shown that colchicine can nonselectively inhibit NLRP3 inflammation by inhibiting the activation of P2X7 receptor or the interaction between NLRP3 protein and ASC.^350,351^ In addition, chloroquine^352^ and curcumin^353^ are also capable of inhibiting NLRP3 signaling. Several investigational agents capable of inhibiting NLRP3 activation, such as tranilast,^354^ dapansutrile (OLT1177, selective inhibitor),^354^ and thiazolo-alkenyl sulfonylurea derivative 7,^355^ are also being considered for COVID-CS treatment as reviewed by Freeman and Swartz.^164^ In addition, some nonselective or selective agents against NLRP3 inflammasome including melatonin (NCT04409522), OLT1177 (NCT04540120), açai palm berry extract (*Euterpe oleracea*) (NCT04404218), and ZYIL1 (selective inhibitor) (NCT04731324) are under investigation.

## Interventions targeting multiple cytokines and pathways

### Intravenous immunoglobulin (IVIg) therapy

IVIg is a natural immunoglobulin pool with a highly diverse antibody network and is administered to superimpose over a patient’s imbalanced repertoire caused by infections.^356^ It has been known for a while that a broad range of natural anti-cytokine autoantibodies such as those against IL-1, IL-6, and IFN-γ can be detected in the IVIg of healthy individuals.^357–360^ Although how the autoantibodies are induced is still poorly understood, it has been demonstrated that many of the anti-cytokine autoantibodies are neutralizing antibodies and may be responsible for the anti-inflammatory effect of IVIg in inflammatory and autoimmune disorders.

The potential efficacy of IVIg therapy was reported in SARS and the 2009 H1N1 influenza pandemic.^361,362^ In a randomized, controlled trial including 84 COVID-19 patients, IVIg treatment did not demonstrate any therapeutic benefits in severe cases; however, a significant positive relationship between the number of days from admission to IVIg treatment and the length of hospitalization was observed,^363^ which indicated the potential clinical benefit of IVIg administration during the early stage of COVID-19. In contrast, Suzuki et al.^364^ reported the potential efficacy of IVIg administration along with mechanical ventilation, methylprednisolone, favipiravir, ivermectin, and tocilizumab therapy in the late phase in an elderly patient with severe COVID-19.

In general, these studies were inconsistent with respect to the timing of IVIg administration, i.e., early phase vs. late phase; therefore, a larger number of clinical trials are warranted.

### Corticosteroid treatment

Corticosteroids are one of the most commonly used anti-inflammatory drugs in the treatment of many inflammatory disorders. They exert immunoregulatory effects by inhibiting the expression of multiple pro-inflammatory cytokines and activation of various immune cells. At the beginning of 2020, corticosteroid treatment in COVID-19 was either contraindicated or not recommended,^365^ because of the statement that no clinical data indicated a benefit from corticosteroid treatment. Moreover, it even increased the mortality and secondary infection rates in SARS-CoV and MERS-CoV.^366^ In March 2020, the RECOVERY trials, one of the largest randomized, controlled trials for COVID-19 treatments including ~15% of all hospitalized COVID-19 patients in the UK, were launched. The dexamethasone arm enrolled 2104 patients receiving a low-to-moderate dexamethasone dose of 6 mg per day for 10 days and the control arm comprised 4321 patients receiving standard care. Compared to the control group, dexamethasone treatment reduced the 28-day mortality by one-third in mechanically ventilated patients and by one-fifth in patients receiving oxygen only, but not in patients with no need for ventilated support.^367^ Therefore, the UK government (https://www.gov.uk/government/news/world-first-coronavirus-treatment-approved-for-nhs-use-by-government/) and the National Institutes of Health in the United States (https://www.covid19treatmentguidelines.nih.gov/dexamethasone/) have authorized the standard use of dexamethasone in hospitalized COVID-19 patients who require oxygen. In addition, a meta-analysis of seven clinical trials showed that corticosteroid treatment was associated with a lower 28-day all-cause mortality in critically ill COVID-19 patients.^225^

Collectively, the clinical benefit of glucocorticoids in COVID-19 treatment is based on the selection of the correct dose, correct patient, and appropriate timing. Several studies have shown the temporal dynamics of viral shedding in SARS-CoV-2.^368–370^ After the replicative peaks, immunopathological factors may play a dominant role in the illness, while active viral replication may play a secondary one. Therefore, the viral load may serve as an indicator to determine the precise time of glucocorticoid treatment. Moreover, in contrast to other agents, dexamethasone is readily available worldwide at a low cost, which is beneficial in developing countries with limited access to health care. However, although corticosteroid improves clinical syndromes in critically ill COVID-19 patients, its impact on CS is still unclear and requires further investigation.

### Traditional Chinese medicine (TCM) treatment

Previous studies have reported the therapeutic effects of several TCMs such as Lizhong Decoction,^371^ Liujunzi Decoction,^372^ and Huanglian Jiedu Decoction^373^ on inflammatory diseases. Recently, TCM treatment has also been shown effective for COVID-19 and contributed substantially to control the pandemic in China. For example, a multicenter, randomized, controlled trial by Nanshan Zhong’ research team showed that Lianhuaqingwen capsule, a repurposed Chinese herb, can ameliorate clinical symptoms and shorten the recovery time in COVID-19 patients with no serious adverse effects.^374^

Several TCMs may play an immunosuppressive role to treat CS via multiple cytokines or pathways related to the CS. Yang et al.^375^ showed that Qingfei Paidu Decoction, one of the most well-known anti-COVID-19 formulae, can defend against COVID-19 by regulating multiple CS-related signaling pathways such as the NF-κB and MAPK pathways and cytokines such as TNF-α, IL-1β, and IL-8. In addition, Dai et al.^376^ conducted a large-scale transcriptional study to evaluate the effects of 578 herbs and all 338 reported anti-COVID-19 TCM formulae on CS-related signaling, by combining high-throughput sequencing-based screening assay with bioinformatics and computer-aided drug design. The results showed that some herbs might inhibit the IL-6 pathway, some, the TNF-α pathway, and some drugs such as Guizhi and Qingfei Paidu Decoction may inhibit both pathways. Together, these studies provide scientific evidence for the administration of TCMs in COVID-CS.

### Blockade of cyclin-dependent kinase 7 (CDK7)

It is challenging to block individual cytokines to achieve the desired clinical benefit given the complicated crosstalk of cytokine signaling during CS.^377^ Thus, developing anti-inflammatory strategies with a wide-spectrum inhibitory effect is urgently required.

CDK7 can regulate cell cycle and gene transcription.^378^ Previous studies have shown that blockade of CDK7 manipulated inflammation resolution by remodeling antitumor immunity^378^ and regulating cytokine secretion.^379^ A recent study by Wei et al.^380^ showed that small-molecule inhibitor, THZ1, mediated the blockade of CDK7 and thus mitigated hyperinflammatory states and CRS caused by CAR T cell therapy. Mechanistically, when CDK7 is blocked by THZ1, various pro-inflammatory genes, especially *STAT1* and *IL1* that are regulated by CDK7/RNA Pol II super-enhancers, are preferentially suppressed at the transcriptional level. This indicates that blockade of CDK7 may be a promising strategy to treat CRS. Considering similar cytokine profiles between COVID-CS and CRS, we can hypothesize that the strategy may also work in COVID-CS.

## Conclusions

Clinical and basic research studies have identified and characterized COVID-CS, which has greatly enhanced our understanding of CS and related immunopathology in COVID-19. A full scenario of COVID-CS is now emerging, and it appears to be much larger in scale and contains more cytokines than the CS recognized in other conditions, and is therefore also more damaging. Although it is still unclear how the virus turns the protective cytokine profile into an inflammatory CS, the cytokines appear to be produced predominantly by innate cells because the lymphopenia was frequently reported in this condition. While the causative efficacy of individual cytokines on the development of certain immunopathogenic parameters in this condition is yet to be fully understood; it is obvious that COVID-CS as a whole is closely associated with the major pathogenic changes of COVID-19. To treat COVID-CS, several biologic interventions specifically targeting inflammatory cytokines or related signaling pathways have been clinically evaluated with promising results and many others are in the pipeline.

In principle, the treatment strategy should be to control ongoing inflammatory cytokine production or activity and resume the host’s homeostasis. However, we still lack safe and effective drugs to control the CS, and clinically, the treatment of CS has been proved difficult for several reasons: (1) many medical doctors are not aware of the condition and hence, clinical diagnosis and treatment guidelines are currently lacking; (2) it is a pharmaceutical challenge to simultaneously target multiple cytokines. It is therefore important to identify and target the key cytokines upstream and the cytokine induction network, or directly target the predominant cytokine-producing cells such as monocytes and macrophages. Alternatively, learning from the host immunoregulatory system and identifying more effective and safer anti-inflammatory factor/cytokines with a wide-spectrum inhibitory effect may provide a better option for therapeutic intervention; IL-37 has been suggested for this purpose;^381–384^ (3) it is difficult to balance CS and protective immunity in infectious diseases, as the appropriate level of inflammatory cytokines is protective against infections and inappropriately targeting inflammatory cytokines may lead to acquired immunodeficiency and subsequent infections; (4) differences among individuals with respect to age, immune status, and other comorbidities may result in virtual differences in the component and scale of the CS and treatment. Therefore, precise treatment is required. Of note, ideally targeting predominant cytokines or molecular pathways in a particular CS condition should be conducted first and in a timely manner.

COVID-19 has taught us a critical lesson regarding how to deal with natural pathogenic enemies. Knowledge and treatment options developed from COVID-CS will be invaluable, not just for this disease but also for other CS conditions.
